# Co-movement between stock markets in advanced economies and Africa in times of uncertainty: A time-frequency domain approach

**DOI:** 10.1371/journal.pone.0334325

**Published:** 2025-11-06

**Authors:** Joseph Emmanuel Tetteh, Peterson Owusu Junior

**Affiliations:** 1 Department of Finance, Central Business School, Central University, Tema, Ghana; 2 Department of Finance, School of Business, University of Cape Coast, Cape Coast, Ghana; University of the Witwatersrand Johannesburg, SOUTH AFRICA

## Abstract

This study examined the co-movement between New York and Shanghai stock markets, and twelve African stock markets, before, during, and after the COVID-19 pandemic. Daily composite indices from January 2016 to March 2023 were used for the study. The study employed the continuous complex Morlet wavelet transform which is best for time-frequency domain in terms of magnitude, direction, and lead-lag in localised linearity, and stationarity. The results revealed notable co-movements between the two advanced markets and some African stock markets. However, considerable number of co-movements between the two advanced markets and most African stock markets were not significant. Furthermore, the study found that the nature of co-movement between advanced and African markets reflects interdependence more than contagion. The results further indicate that, the long-held assertion that African stock markets are resilient to fluctuations in advanced markets during periods of global turbulences if gradually fading away. This study addresses a critical gap in the literature concerning the influence of pandemics on co-movement of markets with a specific focus on co-movement between stock markets in advanced economies and those in Africa. In addition, it departs from previous studies by employing a bivariate wavelet approach which effectively handles non-linearity, non-stationarity, structural breaks and time localization. We recommend that policymakers incorporate both time and frequency characteristics of markets into market regulations and strategies. Investors should employ risk minimisation strategy through the creation of international portfolios between global and emerging African markets to enhance their reward from investments in stocks, albeit with due caution.

## 1. Introduction

The financial landscape of the global economy is characterised by a complex web of interconnected markets, which is often evaluated through the lens of co-movement analysis by academics. Understanding the co-movement between stock markets in developed economies and those in emerging markets, such as African markets, offers valuable insights into the broader impacts of global economic events, including COVID-19 pandemic.

Historically, the co-movement between advanced and African stock markets has been relatively weak or non-existent. This phenomenon has been attributed to various factors, including the differing levels of market maturity, liquidity, regulatory environments, and underlying economic structures between these regions [1]. Advanced markets, often characterised by higher liquidity, greater investor participation, and more stringent regulatory frameworks, tend to respond differently to global shocks compared to African markets [2].

The global COVID-19 outbreak presents a unique context in which to examine how these markets co-move during a global crisis. The pandemic caused unprecedented disruptions in economic activities and financial market operations. While several studies on the effect of the COVID-19 pandemic on financial markets, highlight upsurge in volatility and breakdown of the conventional relationships between various markets [3], the co-movement between advanced and African stock markets remain under-explored. Given unique characteristics of African markets, including lower levels of international investment and diversification, it is plausible that these markets were partially insulated from the immediate impacts of the pandemic as observed in more integrated and developed markets [4].

Empirical evidence suggests that the initial phases of pandemics witness the decoupling of emerging markets from their advanced counterparts. For instance, while the major stock market indices in advanced economies suffered sharp declines, many African stock markets did not exhibit the same level of drastic movements [5]. This discrepancy may reflect African economies’ relatively lower exposure to global trade and finance, as well as differences in the timing of the pandemic’s spread and the policy responses across regions.

The degree of co-movement among African stock markets has been the subject of recent empirical research. Different degrees of dependency may offer opportunities for risk reduction and the benefits of international portfolio diversification strategies, which has increased scholarly interest in this subject area [6]. Research indicates that although some linkages have emerged between African and international stock markets particularly during periods of global crises, their overall degree of integration is low [7]. This offers both domestic and foreign investors potential diversification advantages especially as African stocks are recognized for offering higher returns [8].

The co-movements of stock markets and their vulnerability to shocks and uncertainties in the global economy suggests a high probability that the COVID-19 pandemic significantly influenced the co-movement pattern between African stock markets and those of developed economies [9,10]. Moreover, research suggests that co-movements of stock markets tend to intensify during crises, including epidemics/pandemics [11].

It is significant to highlight the paucity of research on the effects of pandemics on the co-movement of the stock markets between advanced economies and Africa. Majority of previous studies paid limited attention to the relationship between markets in advanced economies and African, even though some scholars observed low levels of interdependence between the two sets of markets, which point to possible advantages for international portfolio diversification and risk mitigation [6]. This suggests that some African stock markets and those in advanced economies are moving in tandem to some extent, which underscores the growing relevance of this research area.

This study investigates stock market co-movement between two stock markets in advanced economies, namely New York stock Exchange (NYSE) and Shanghai Stock Exchange (SSE), before, during, and after COVID-19 period.

Globally, the NYSE is widely regarded as the biggest and most impactful stock market. It reflects the financial dynamics of the developed countries, particularly the US, which is a dominant player as far as shaping of global economic and financial trends are concerned. Through trade, foreign direct investment, aid, and multinational institutions, the USA has significant impact on African markets [12,13]. In terms of market value and trade volume, the SSE is among the biggest market places globally, despite being an emerging market. SSE embodies the increasing sway of China which is widely regarded as the economic superpower of the East. t. China has become Africa’s main bilateral trading partner, and a major investor with respect to energy, mining, and infrastructure. [14,15]. The SSE was chosen for the research because of China’s increasing economic interest in Africa, while the NYSE was chosen because of its overall impact on international markets, including those in Africa. Both are vital for assessing global market spillovers to African markets, especially in times of uncertainty, because they collectively offer a fair perspective on both Western and Eastern financial systems

It has been reported that during economic downturns, financial markets experience considerable instability as a result of rapid information transmission. This phenomenon has increasingly drawn the interest of researchers in co-movement research [16]. In order to make informed decisions, investors frequently seek out and analyse information in the financial market [17].

By the employment of bivariate wavelet analysis, this study deviates from the use of conventional methods such as panel regressions, correlation analysis, vector autoregression, error correction models, and GARCH models that do not capture both time and frequency dimensions in financial data, and in addition fail to sufficiently address the issues of non-stationarity and structural breaks [18]. The bivariate wavelet technique allows for an in-depth analysis of how COVID-19 influenced stock market relationships across different time horizons in contrast to multivariate approaches [19]. It disaggregates time series data into several wavelet time scales, and effectively handles non-linearity, non-stationarity, structural breaks and time localization [20]. Choosing the right statistical approach is essential for assessing the dynamic relationship between stock markets, especially in times of uncertainties. Time-varying co-movement is frequently captured by conventional models like Time-Varying Parameter Vector Autoregression (TVP-VAR) and Dynamic Conditional Correlation GARCH (DCC-GARCH) [21]. However, these conventional models tend to be limited in their ability to capture non-linear, multi-scale financial dynamics, since they primarily function in the time domain and often assume stationarity or linearity [22]. By providing a time-frequency breakdown of co-movement, wavelet coherence analysis allows researchers to evaluate how connections change over time and over various investment horizons [23]. This is particularly crucial when examining how shocks, such as epidemics, affect short-, medium-, or long-term co-movement distinctly. As a result, the wavelet coherence provides a thorough understanding of intermarket dynamics, which makes it suitable for research on diverse markets, including those in advanced economies and Africa, where co-movement may differ by event and scale [24,25].

This method offers a robust and comprehensive framework for examining the link between stock markets and assessing the potential influence of the COVID-19 pandemic. It provides a dynamic examination of market co-movement at different time frequencies, offering deeper insights than traditional methods.

The paper is organised into five Sections. The second Section reviews previous literature on the subject area, the third Section presents detailed methodology employed in the study, while the fourth Section presents the results and discussion. The conclusion and implications to the study are captured in the final Section.

## 2. Literature review

### 2.1 Theories underpinning stock market co-movement

Several theories such as efficient market hypothesis (EMH), information spill-over effect, contagion theory, economic integration theory, globalization and financial liberalization, behavioral finance theory, information transmission theory have been employed to explain the degree to which indices or prices in different stock markets move in tandem. The EMH argues that the information available at a given moment in time justifies asset prices in the market [26].

Behavioural finance theory embodies the way and manner in which subjective variables, such as investor preferences for asset allocation and the herding effect have a significant impact on the connections between securities markets [27]. Investor behaviour evolves, especially during turbulent periods like the COVID-19 pandemic. Time-based behaviour is taken into consideration in both market pricing and investors’ asymmetrical judgments since markets are not isolated systems. Furthermore, markets may move more in tandem when news is overreacted to and underreacted to than would be supported by fundamentals alone [28,29]. Investors also tend to overestimate recent losses or crisis news due to availability and recency biases. Even among markets that were previously uncorrelated, this might have the impact of spreading and speeding up market fluctuations [30,31].

Three other hypotheses, the Heterogeneous Market Hypothesis (HMH) [32], the Competitive Market Hypothesis (CMH) [33], and the Adaptive Market Hypothesis (AMH) [34]—all lend further support to the discussion of stock market co-movement. The EMH assumes that investor rationality is constant, regardless of time or situation. In contrast, some scholars argue that investor rationality and irrationality vary over time and among investors [35]. In addition, the AMH emphasizes examining investor behavior over smaller subsamples rather than the full sample, pointing out varying degrees of market efficiency. The HMH contends that various economic players take into account a range of time horizons, risk preferences, and return expectations, and that their decisions are influenced by both recent and historical news. Moreover, during challenging market conditions, investors’ search attempts are impeded, which further obstructs the flow of information between market players. The methods used in this work, which provide a thorough dynamic investigation of the correlations across numerous frequencies and probable causative structures, are therefore supported by the AMH, HMH, and CMH.

These investor-driven complications are thought to exist across a broad spectrum of markets, each characterised by different levels of risk and return, both in theory and in practice. This observation reinforces the fundamental principle of portfolio diversification, which aims to optimise returns while minimising risk exposure. These markets are well-known for their unique qualities as well as the return and competitive risks they offer. Furthermore, traditional investors may or may not diversify into asset classes that correspond to the overall market. Assets that effectively meet the investor’s needs, particularly in times of volatility are commonly referred to as safe heavens [6].

The core concept of portfolio diversification has been extensively examined in various studies aiming to mitigate risk and secure favourable returns, particularly in the context of financial downturns and health crises [36,37]. According to portfolio diversification theory, uncorrelated or weakly correlated assets can lower portfolio risk for a given expected return [38]. This suggests that low market correlations are more advantageous to investors. Due to their limited integration with international markets, African markets have traditionally provided diversification benefits [39]. However, because of global investor herding, market correlation grows during crises, diminishing the potential benefits of diversity [40].

Market integration involves the extent to which distinct financial markets move in tandem and exhibit a common set of risk variables. Theoretically, regardless of location, assets with identical risk characteristics should provide comparable returns in a fully integrated market [41,42]. It emphasizes that, market integration occurs when steps are taken to eliminate obstacles to global financial flows and investors may easily diversify their holdings across national borders. In general, trade openness, financial liberalization, institutional quality, and macroeconomic convergence are factors that propel market integration [43]. However, it is expedited by programs like the AfCFTA and during uncertain periods like the COVID-19 era [44]. Because of capital constraints, illiquidity, and low investor engagement, many African markets continue to be poorly segmented [45].

Theories of co-movement of stock market provide valuable frameworks for understanding the interconnectedness of global financial markets. These theories, which emphasize factors such as investor behavior information transmission, and economic integration, offer critical insights into the mechanisms underlying market synchronization. In practice, multiple theories often interact, shaping the co-movement patterns observed in real-world financial markets.

### 2.2 The empirical review of stock market co-movement

Majority of co-movement of financial markets are concentrated on adanced economies. Baruník and Křehlík (2018) [46] for instance introduced a new framework for measuring connectedness among financial variables that arise as a result of heterogeneous frequency reaction to shocks in U.S. financial markets. Connectedness observed at lower frequencies suggests that shocks are more persistent and transmission extends over a long period. Although a vast body of literature has examined the effects of global crises on advanced and some emerging markets, little attention has been given to the co-movement between African and global stock markets during such crises.

The links between advanced markets, particularly between the US and other developed stock markets, are the main focus of the majority of research conducted thus far on the co-movement of stock markets [46,47]. There has been little co-movement between advanced stock markets and African stock markets. Several factors have been cited to explain this weak co-movement, including disparities in market maturity, liquidity, efficiency, and the level of economic integration with the global financial system. Some studies for instance have found that African stock markets have low levels of market integration with global markets, largely due to structural inefficiencies and low participation from foreign investors [1]. Anyikwa and Le Roux (2020) [48] investigated the extent of market integration and contagion between four developed countries (the United States, France, Germany, and the United Kingdom) and seven African stock markets during the 2008 global financial crisis and the Eurozone’s sovereign debt crisis. Their findings revealed minimal evidence of market integration between African and developed markets. Furthermore, Atenga and Mougoué (2021) [49] examined the volatility and returns of financial markets in seven African stock markets from the US, UK, Brazil, China, France, Germany, Japan, Mexico, and Russia. Although significant spillovers did occur during the global crises of 2008 and 2012, their analysis showed a very weak transmission of external shocks to African stock markets.

Nonetheless, some studies have argued that there is the tendency for increased integration with global markets as African economies undergo financial liberalization and economic reforms [42].

A small number of scholars have undertaken empirical study on co-movement between Western and African markets as a result of calls for closer linkages between the African stock markets and other markets [50,51].

Africa’s emerging markets therefore have good chances for diversification, especially in light of the periodic spikes in market volatility worldwide. More and more studies need to be conducted to examine the cross-market linkages and co-movements between African and other markets in order to evaluate the validity of this assertion [49].

### 2.3 Co-movement during global uncertainties

#### 2.3.1 *Financial crises.*

The notion that financial crises can boost interconnectedness by impacting financial markets internationally has been substantiated by empirical research [11,31]. According to Wang et al. (2017) [11], co-movement between European markets and other international stock markets increased during the global financial crisis of 2008 but declined sharply thereafter. Their findings have been reaffirmed by BenSaida and Litimi (2021) [52]. In addition, Escribano and Iniguez (2020) [53] discovered that the Brexit referendum and the resulting increase in unpredictability caused financial contagion that had a major effect on several European and global economies.

The global financial crisis had a varied of effects on African markets. While certain African markets exhibited increased linkage with global markets during the crisis, others remained relatively insulated [54].

#### 2.3.2 *Epidemics/pandemics.*

The impact of pandemics and epidemics on stock market co-movements has received relatively limited scholarly attention, even though recent developments in global capital markets have significantly expanded the breadth of knowledge in co-movement in academic circles. According to most studies, the financial markets were mostly unaffected by plagues like Zika, SARS, Ebola, and swine flu [55,56].

Despite the extensive body of research on the COVID-19 pandemic, relatively limited attention has been devoted to its impact on the interdependence between advanced and African stock markets, compared to the substantial focus on its effects on stock market returns and volatility within African markets [57,58].

Fortunately, a small number of research have looked at how advanced and African markets move together. Omane-Adjepong et al. (2020) [19] for instance, found that the magnitude of pairwise series co-movements was significantly higher during the COVID-19 period compared to the pre- and post- pandemic periods. This finding has been reiterated by Owusu Junior et al. (2024) [8] in their study on co-movements between African stock markets. These results give credence to the hypothesis that stock market co-movements are strongly influenced by crises. The chaotic information flow caused by the COVID-19 pandemic to twenty-seven foreign financial markets was examined by Owusu Junior, Adam et al. (2021) [59] between December 31, 2019, and April 18, 2021. Their results support the notion that there is greater opportunity for short- to medium-term diversification at this time. This is consistent with previous research by Tweneboah et al. (2019) [18], who African stock markets offer fewer diversification opportunities at lower frequencies than at higher ones, implying stronger co-movement over the long term and weaker co-movement in the short term.

The literature reviewed so far suggests a notable gap in examination of co-movements between African and global stock markets, particularly during periods of pandemics. This significant gap is what this study seeks to fill.

### 2.4 Contagion versus interdependence

Distinguishing contagion from interdependence is central to the analysis of cross-market co-movements. Yang et al. (2016) [43] opined that by examining shifts in coherencies, one can distinguish between true contagion and simple dependency. According to their definition, contagion refers to the quick increase in correlations that occurs at low frequencies (medium- to long-term) at specific time periods. This description is consistent with Forbes and Rigobon’s (2002) [44] idea of contagion, as sudden spikes in correlations happened during crisis situations. According to their definition, contagion is a shift in the way that different asset classes are transmitted, such as a notable increase in cross-market correlation following a shock or crisis.

Bekaert et al. (2014) [60] expanded this further by propounding out a more complex framework based on global factor models. They define contagion as the co-movement that remains after taking regional and global causes into consideration. Therefore, it embodies an unusual increase in market connection that cannot be explained by underlying causes or preexisting dependency, especially during times of crisis. This concept is helpful in determining whether observed market movements are a result of cross-border transmission of idiosyncratic risks or are responses to common external shocks. It should be noted that physical exposure and asymmetric information are the two main processes that commonly drive contagion. Therefore, a shock experienced in one market may cause shocks in other markets, irrespective of the underlying fundamentals [40].

Conversely, interdependence is defined as a notable increase in the correlation between two markets that remains stable over time [61]. Interdependence is therefore perceived to be high correlations between markets that are stable over time, including during crises, and are typically driven by underlying economic and financial fundamentals [40]. Additionally, interdependence was described by Beirne and Gieck (2012) [62] as the average relationship between asset classes during a specific time period. Co-movement is more frequently brought on by shared factors or increasing market interdependence than by shocks spreading directly.

## 3. Methodology

### 3.1 Data collection and processing

The study used daily data from two advanced markets, the Shanghai Stock Exchange (SSE) and the New York Stock Exchange (NYSE), as well as daily composite index data from twelve African stock markets: Botswana, Mauritius, Egypt, Morocco, Tunisia, Ghana, Kenya, Nigeria, South Africa, Tanzania, Zimbabwe, and Zambia. These markets were selected for this study taking into consideration availability of data and regional representation. At least two stock markets were selected from the four main regions of Africa, namely North, West, East and Southern Africa. The data covered the period from January 1, 2016, to March 31, 2023. Pre-COVID-19 period (January 1, 2016 to February 29, 2020), COVID-19 period (March 1, 2020 to February 28, 2021), and post-COVID-19 period (March 1, 2021 to March 31, 2023) are the three sub-periods into which the data is divided. These sub-periods were determined based on three factors, namely actual market reactions, WHO declarations, a fundamental examination of pandemic-related data from the World Health Organization (WHO) and previously published works [8].

The study captured different market situations in reaction to the COVID-19 pandemic’s global effects because of the use of daily data over a long-time span. The long-time horizon enables the analysis to capture different market situations in reaction to the COVID-19 pandemic’s major worldwide effects.

Data on composite stock market indices was obtained from EquityRT (https://equityrt.com/), while data on COVID-19 was provided by Our World in Data (https://ourworldindata.org/).

### 3.2 Bivariate wavelet coherence

The bivariate wavelet analysis methodology serves as a formidable tool for examining the relationships between two time series across multiple time scales. This method decomposes the time series into wavelets, which provide information about both the frequency and location (time) of the signal features. In this study, it provides a robust framework for examining the co-movement between two stock markets in advanced economies, namely New York and Shanghai stock markets, and twelve African stock markets.

According to Sang (2013) [63], a wavelet is a tiny wave that can be stretched over time to expose frequency components hidden in complicated signals. Time and frequency localize the null mean of the wavelet function. The wavelet transform can be written as follows if the time scale and time position parameters are t, q, and τ, respectively, and factor 1/√q is the normalization component that guarantees unity in variance:


$$\Phi_{\tau ,q}(\text{t})\;=\;\frac{\text{1}}{\sqrt{q}}\Phi \left(\frac{t-\tau }{q}\right)$$

Although financial studies employ various types of wavelet techniques, the Morlet Wavelet—an offspring wavelet of the parent wavelet, is most suited for this research. It is helpful because of its strong localization features within the Gaussian fold, both in the frequency and temporal domains. The Morlet wavelet can be stated as:


$$\Phi_{\text{0}}(\gamma )=\;\frac{\text{1}}{\sqrt[{\text{4}}]{\pi }}e^{i\eta w_{\text{0}}}.\frac{\text{1}}{\sqrt{e^{\eta^{\text{2}}}}}$$

where the temporal and dimensionless central frequency parameters are represented by η = t/q and w_0_, respectively, and the negative fourth root is a normalization term. Setting w_0_ to 6 yields the optimum time-frequency localization, according to Rua and Nunes (2009) [24]. A discrete or continuous transformation is necessary for wavelet analysis. The Continuous Wavelet Transform (CWT) is the proper transformation needed for the continuous data set {x(t), 1,..., n}. The procedure seeks to maintain the data series’ energy for upcoming power spectrum research. The CWT’s resultant function with respect to (t) is:


$$\text{Vx}(\tau ,\text{ q})\;=\frac{\text{1}}{\sqrt{q}}\int_{-\infty }^{+\infty }\text{x}(\text{t})\;\Phi *\left(\frac{t-\tau }{q}\right)dt\;$$

where * denotes a complex conjugate. The energy density of the signal in the frequency-time domain is represented by the Wavelet Power Spectrum, or |Vx| 2, if Vx(τ, q) is the signal {x(t), 1,..., n}. Wavelet coherence (WC), wavelet phase-difference (WPD), and cross-wavelet power (CWP) are the most effective techniques for determining and quantifying the relationship between two different continuous wavelet transforms (Va and Vb). For the signals x(.) and y(.), the CWP as illustrated in Hudgins et al. (1993) [64] shows the local covariance of the two signals at various scales and frequencies. This can be stated as:


Vab = |VaVb|

We determine the wavelet coherence (WC) or correlation between two time series (composite stock index pairs) in the time-frequency domain [42].


$${\text{R}}^{\text{2}}(a,b)=\;|\text{S}({\text{a}}^{-\text{1}}{\left|{\text{W}}_{\text{XY}}(a,b)\right)|}^{\text{2}}$$

$$S(a^{-\text{1}}{\left|{\text{W}}_{\text{X}}(a,b)\right|}^{\text{2}})\;S(a^{-\text{1}}{{\text{W}}_{\text{Y}}(a,b)|}^{\text{2}})\;$$

where S is a temporal and scale smoothing operator. A wavelet coherence could be interpreted as a localised correlation in the temporal frequency domain. A wavelet coherence (R^2^(a,b)), like the conventional correlation measure, is a number between 0 and 1, with values around 1 suggesting strong coherence and a value of zero (0) indicating no link. This is helpful in locating areas of the time-frequency space, where there is a significant correlation between the two-time series.

For every time series, the wavelet transform is computed independently [65,66]. The wavelet transform of a time series X(t) using a wavelet ψ is defined as:


$${\text{W}}_{\text{X}}(a,b)\;=\int \text{X}(t)\psi^{*}\frac{\left(t-b\right)}{a}dt$$

where *a* is the wavelet function’s scale, *b* is its translation, and ψ* stands for its complex conjugate.

Grinsted et al. (2004) [67] apply the CWT to each stock index’s log return series. When two time series, X(*t*) and Y(*t*), are wavelet transformed, the result is:


$${\text{W}}_{\text{XY}}(a,b)\;=\;{\text{W}}_{\text{X}}(a,b){\text{W}}_{\text{Y}}(a,b)$$

At various timeframes and scales, W_XY_(a,b) provides insights into the common power and phase relationship between the two time series. The common power is shown by the magnitude ǀW_XY_(a,b)ǀ.

Madaleno and Pinho (2012) [68] first introduced the wavelet phase difference (WPD) function, which sheds light on the lead-lag connection between the two-time series. At each time-frequency point, the phase difference between the two series is computed as follows:


$$\varphi_{\text{XY}}(\text{a},\text{b})\;=\text{ arg}({\text{W}}_{\text{XY}}(\text{a},\text{b}))$$

where ϕXY(a,b) is the phase difference. The series move together when the phase difference is zero, and one series leads or lags the other when the phase difference is ± π/2. The signal which is ahead of the other is indicated by the sign of the phase [69].

Phase information is essentially a local measurement of the phase delay, which depends on both frequency and time, between two signals. The arrow directions are examined in order to interpret the phase-difference between the signals. The relative phase (lead/lag) relationships between the two signals are visually indicated by arrow orientations. Right-pointing arrows suggest that the two signals are in phase, whereas left-pointing arrows suggest that they are out of phase (anti-phase). Specifically, if the arrows point right and upward, the first variable is leading in phase; if they point right and downward, the second variable is leading in phase. In contrast, it is suggested that the second variable is leading out of phase if the arrows point left and up, and that the first variable is leading out of phase if the arrows point left and down [69]. The direction of arrows and their implications are illustrated in Table 1.

**Table 1:** Lead-lag coherence relationship.

Direction	Implication
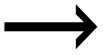	Both markets move together (positive relationship)
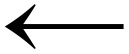	Both markets move together (negative relationship)
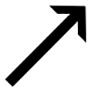	First market leads the second (positive relationship)
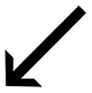	First market leads the second (negative relationship)
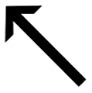	Second market leads the first (negative relationship)
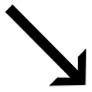	Second market leads the first (positive relationship)

In every pair, NYSE and SSE are considered the first market and the African markets the second market.

In the time-frequency wavelet domain, coherence estimates the local correlation between two time series. A strong correlation at a particular time and frequency is indicated by high coherence (near to 1), whereas a poor correlation at a given time and frequency is indicated by low coherence (close to 0).

High covariance regions in the time-frequency domain are found by using the cross-wavelet transform (CWT), which examines the shared power between two time series. With the x-axis usually indicating time and the y-axis representing frequency or scale, the Coherence Spectrum plots coherence values with time and frequency.

Nonetheless, it can be computationally challenging to analyze time series data using all the wavelet coefficients. It is imperative to state that CWT is still widely used in finance research despite this difficulty because it effectively examines how several periodic components of a signal change over time, both independently and in relation to one another.

The bivariate wavelet analysis methodology thus offers a comprehensive framework for examining the co-movement between advanced and African stock markets. By capturing both time-domain and frequency-domain characteristics, this approach provides deeper insights into the dynamic interactions and underlying factors influencing market behavior.

### 3.3 Data and preliminary analysis

**Fig 1:**
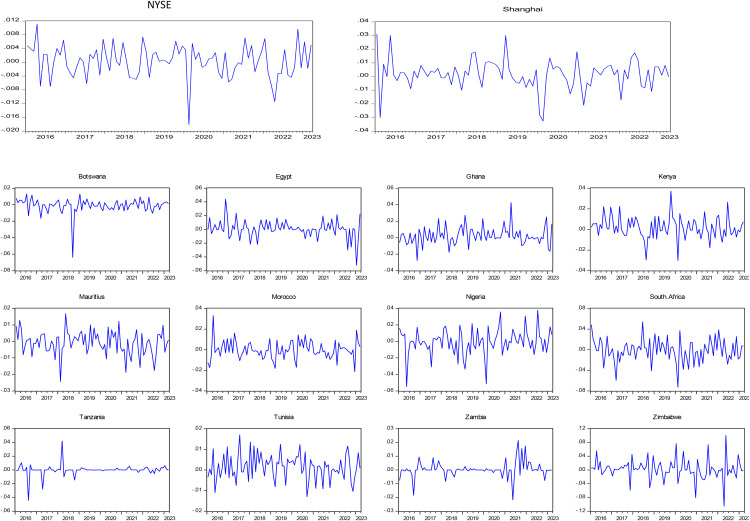
Returns series plots for the markets over the study period.

The data was separated according to real market data and data relating to the pandemic. The categorization was made because statistical tools generally can produce results that do not align with the dynamics of the market. To ensure stationarity, daily closing prices were transformed into log returns. The daily returns were computed by employing the formula: *rt = ln (Pt Pt − 1)*. The return which is continuously compounded is represented by *rt* while the current and previous index values are *Pt* and *Pt − 1* respectively. To enable cross-market comparability, the log returns were normalized to have a zero mean and unit variance. Fig 1 shows the returns on the stock market over time, with notable resemblance in the variations, especially around 2020 with a few markets notably Tanzania showing less impact of the pandemic on their performance. Fig 1 reveals that negative trends can be attributed to the COVID-19 pandemic’s effects. Strikingly, there is a similarity in the variations, especially during the COVID-19 period.

### 3.4 Descriptive statistics

The stock indexes of the advanced markets and that of the selected African markets are summarized in Table 2. The majority of African nations have mean values that are approximately zero, indicating relatively low average return over the analyzed period.

**Table 2:** Summary statistics of selected African and Advanced stock market indexes.

Stock Index	Obs	Mean	Std. Dev.	Min	Max
Zimbabwe	1,871	0.00014	0.07746	−2.74491	0.1839185
Zambia	1,871	0.00018	0.007048	−0.09214	0.0557497
Tunisia	1,871	-9E-05	0.006871	−0.04866	0.0258447
Tanzania	1,871	-4E-05	0.012262	−0.21396	0.2341448
South Africa	1,871	8.4E-05	0.019012	−0.12346	0.0834069
Nigeria	1,871	−0.0003	0.015233	−0.2897	0.0674825
Morocco	1,871	-3E-05	0.009065	−0.09918	0.0503132
Mauritius	1,871	-7E-05	0.00866	−0.10697	0.0996306
Kenya	1,871	−0.0002	0.009327	−0.06539	0.0438118
Ghana	1,871	−0.0004	0.011394	−0.09746	0.1824199
Egypt	1,871	−0.0002	0.018638	−0.38223	0.1028852
Botswana	1,871	−0.0003	0.005995	−0.06376	0.0381851
NYSE	1,873	-8E-05	0.011542	−0.085	0.0560000
Shanghai	1,873	-8E-05	0.011541	−0.08473	0.0555562

Markets such as Zimbabwe and Zambia report slightly positive means (0.00014 and 0.00018, respectively), while others like Tunisia, Nigeria, and Ghana show negative average returns. Zimbabwe exhibits the highest standard deviation (0.07746), reflecting significant volatility, while Botswana has the lowest (0.005995), indicating more stable returns. The maximum values, such as Tanzania’s 0.2341448, suggest occasional sharp positive returns, contrasting with severe downturns like Egypt’s minimum of −0.38223. Both the NYSE and Shanghai indices showed negative mean returns, implying marginally negative average returns over the analysed period. Their standard deviations are identical (0.011542), suggesting a similar level of volatility. However, their maximum and minimum values indicate that neither index experienced extreme fluctuations compared to volatile markets like Zimbabwe or Egypt. Thus, the African stock markets exhibit greater diversity in performance and volatility than their global counterparts, highlighting the unique dynamics and risks associated with each region.

### 3.5 Unit root test

The results of the Augmented Dickey-Fuller (ADF) unit root test (Table 3) indicate that all the analysed stock indices are stationary. This is evidenced by test statistics for each market being significantly lower (more negative) than the critical value of −2.86 at the 5% level.

**Table 3:** Unit root test – Augmented Dickey-Fuller.

Stock market	Lag length	Test statistic	Critical value (5%)	P-value	Decision
Zimbabwe	0	−65.367	−2.86	0.000	Stationary
Zambia	0	−42.389	−2.86	0.000	Stationary
Tunisia	0	−37.086	−2.86	0.000	Stationary
Tanzania	0	−45.905	−2.86	0.000	Stationary
South Africa	0	−37.56	−2.86	0.000	Stationary
Nigeria	0	−39.471	−2.86	0.000	Stationary
Morocco	0	−34.864	−2.86	0.000	Stationary
Mauritius	0	−38.472	−2.86	0.000	Stationary
Kenya	0	−26.21	−2.86	0.000	Stationary
Ghana	0	−33.383	−2.86	0.000	Stationary
Egypt	0	−41.561	−2.86	0.000	Stationary
Botswana	0	−34.619	−2.86	0.000	Stationary
NYSE	0	−43.98	−2.86	0.000	Stationary
Shanghai	0	−43.982	−2.86	0.000	Stationary

The results of the Phillips-Perron (PP) unit root test (Table 4) show that all stock indices are stationary. The null hypothesis of the unit root test is rejected since the test statistics for each market are significantly below the critical value of −2.86 at the 5% level, and the p-values are all 0.000. This result confirms that the time series for each stock market does not exhibit a unit root and is instead mean-reverting, suggesting consistent statistical properties over time. In addition, the results conform with those of the Augmented Dickey-Fuller test, thereby validating the stationarity of the data.

**Table 4:** Unit root test – Phillip Perron.

Stock market	Lag length	Test statistic	Critical value (5%)	P-value	Decision
Zimbabwe	7	−58.824	−2.86	0.000	Stationary
Zambia	7	−42.295	−2.86	0.000	Stationary
Tunisia	7	−37.101	−2.86	0.000	Stationary
Tanzania	7	−48.8	−2.86	0.000	Stationary
South Africa	7	−37.554	−2.86	0.000	Stationary
Nigeria	7	−39.956	−2.86	0.000	Stationary
Morocco	7	−34.84	−2.86	0.000	Stationary
Mauritius	7	−38.524	−2.86	0.000	Stationary
Kenya	7	−26.015	−2.86	0.000	Stationary
Ghana	7	−33.787	−2.86	0.000	Stationary
Egypt	7	−41.383	−2.86	0.000	Stationary
Botswana	7	−34.507	−2.86	0.000	Stationary
NYSE	7	−44.064	−2.86	0.000	Stationary
Shanghai	7	−44.068	−2.86	0.000	Stationary

### 3.6 Correlation test

The NYSE and SSE are weakly correlated (0.196), reflecting weaker relationship between these two global markets (Table 5). Correlations between them and African market indices remain moderate or low, with the South Africa recording the highest correlation coefficients of 0.2724 and 0.2720 with NYSE and Shanghai, respectively. This shows that African markets, in general, are less connected to global markets, a feature which may be attractive to international investors pursuing diversification opportunities.

**Table 5:** Pairwise Correlation Matrix.

Stock Index	Zimbabwe	Zambia	Tunisia		Tanzania	S. Africa	Nigeria	Morocco	Mauritius	Kenya	Ghana	Egypt	Botswana	NYSE	SSE
Zimbabwe	1.000														
Zambia	0.030	1.000													
Tunisia	0.017	0.007		1.000											
Tanzania	0.003	0.004		−0.022	1.000										
S. Africa	−0.001	−0.020		0.222	−0.023	1.000									
Nigeria	−0.004	0.021		0.036	−0.002	0.083	1.000								
Morocco	0.052	−0.014		0.223	−0.029	0.222	0.048	1.000							
Mauritius	0.016	−0.016		0.163	−0.027	0.170	0.081	0.151	1.000						
Kenya	−0.015	−0.005		0.033	0.004	0.055	0.126	0.085	0.140	1.000					
Ghana	0.003	0.064		0.035	−0.005	0.028	0.012	0.077	0.034	0.056	1.000				
Egypt	0.003	−0.019		0.026	−0.035	0.097	0.036	0.178	0.104	0.062	0.046	1.000			
Botswana	−0.004	0.032		0.236	−0.025	0.620	0.018	0.186	0.146	0.002	0.018	0.021	1.000		
NYSE	−0.028	0.001		0.028	0.020	0.272	0.033	0.103	0.135	0.089	0.027	0.068	0.114	1.000	
SSE	−0.030	0.023		0.032	0.039	0.310	0.133	0.093	0.112	0.090	0.030	0.071	0.111	0.196	1.000

The correlation coefficients between African markets are generally low, suggesting weak link between the stock markets. The only strong result is the correlation between Botswana and South Africa which reports a positive correlation of 0.6201, indicating strong integration between the two markets. The results suggest diversification opportunities not only between the advanced markets and African markets, but also between African markets as indicated by Owusu Junior et al. (2024) [8]

## 4. Findings and discussions

### 4.1 Bivariate for pre-COVID-19 period

**Fig 2:**
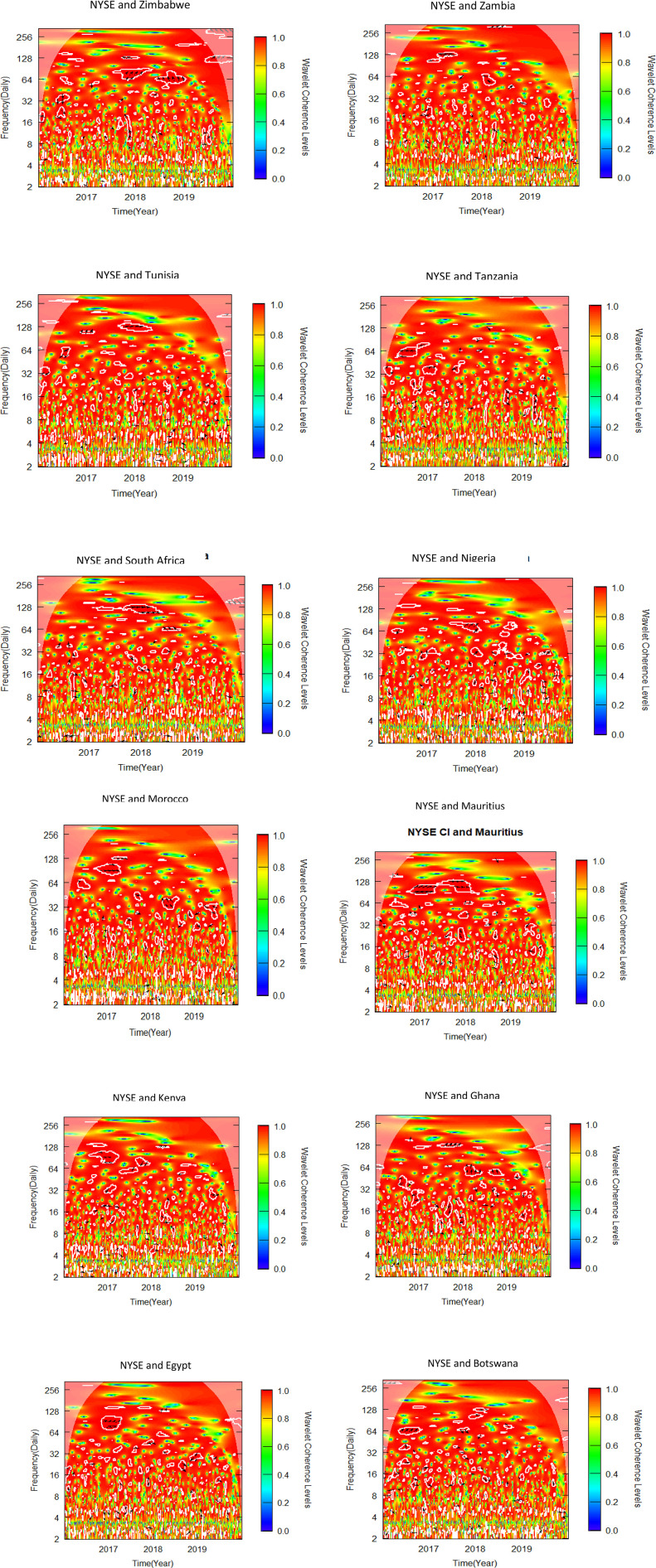
Bivariate wavelet coherence for pre-COVID-19 period (NYSE and African stock markets).

**Fig 3:**
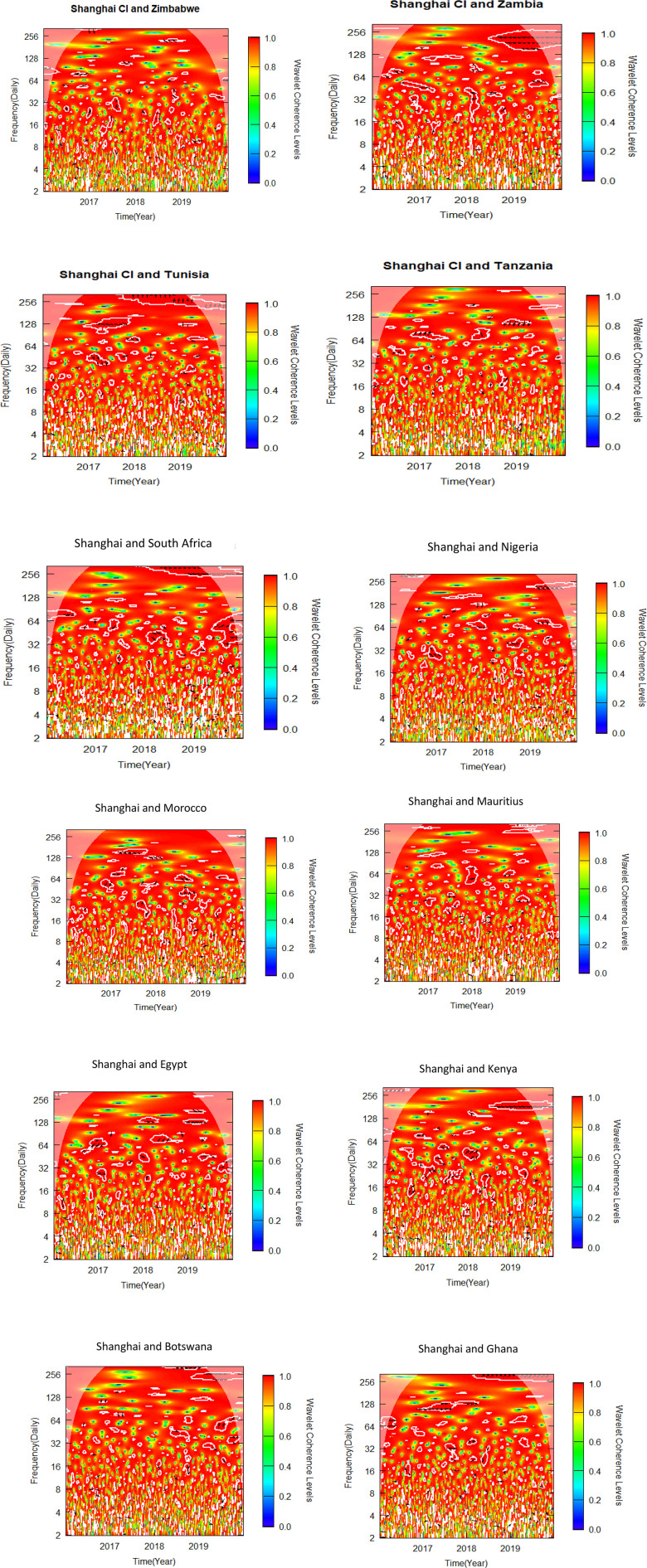
Bivariate wavelet coherence for pre-COVID-19 period. (Shanghai and African stock markets).

**Fig 4:**
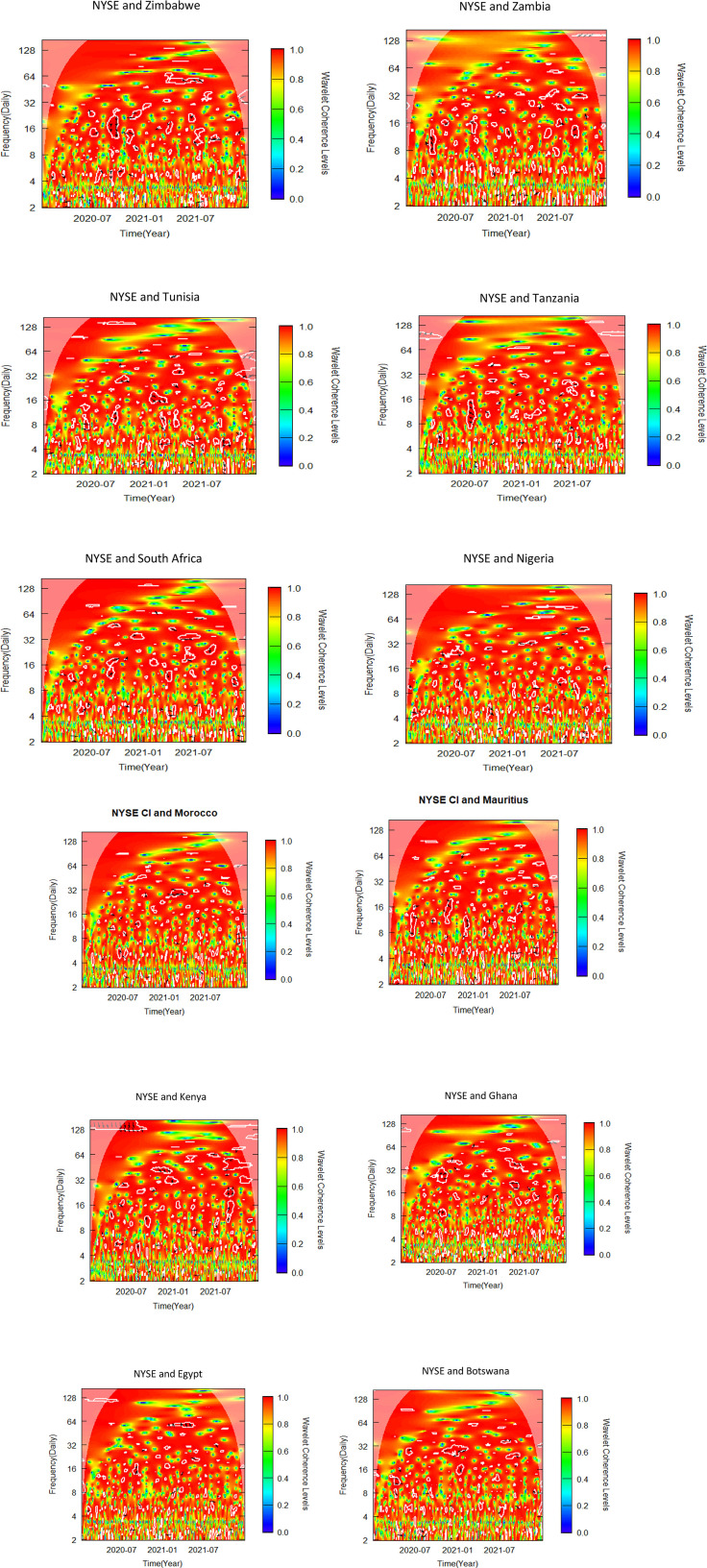
Bivariate wavelet coherence for COVID-19 period (NYSE and African stock markets).

**Fig 5:**
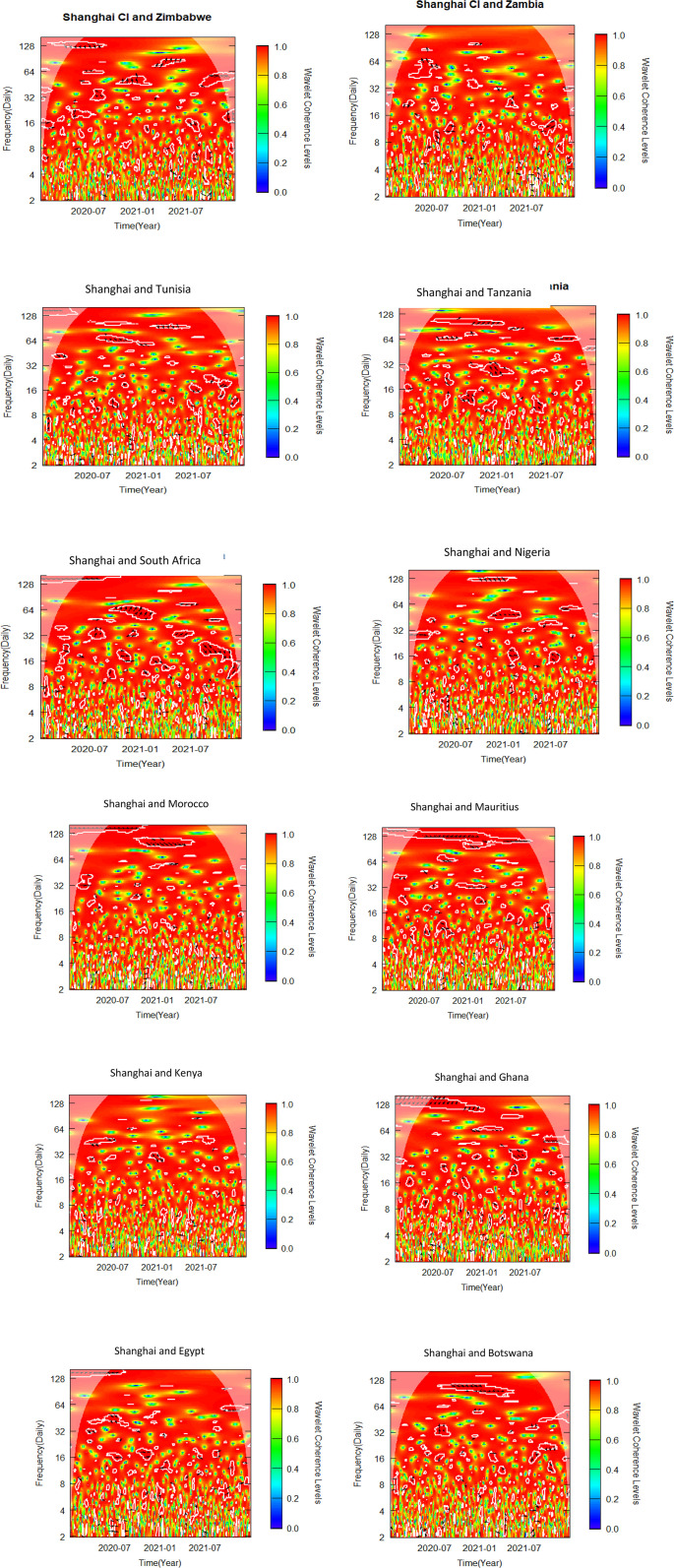
Bivariate wavelet coherence for COVID-19 period (Shanghai and African Stock markets).

**Fig 6:**
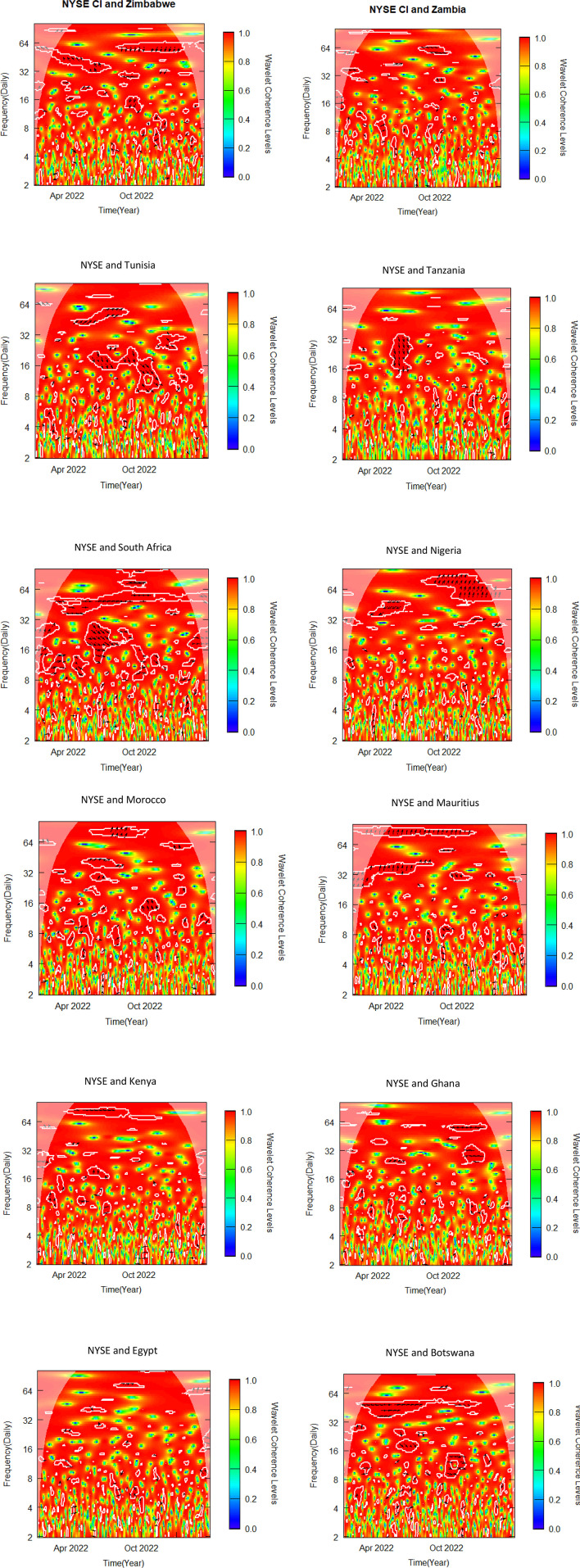
Bivariate wavelet coherence for post-COVID-19 period (NYSE and African stock markets).

**Fig 7:**
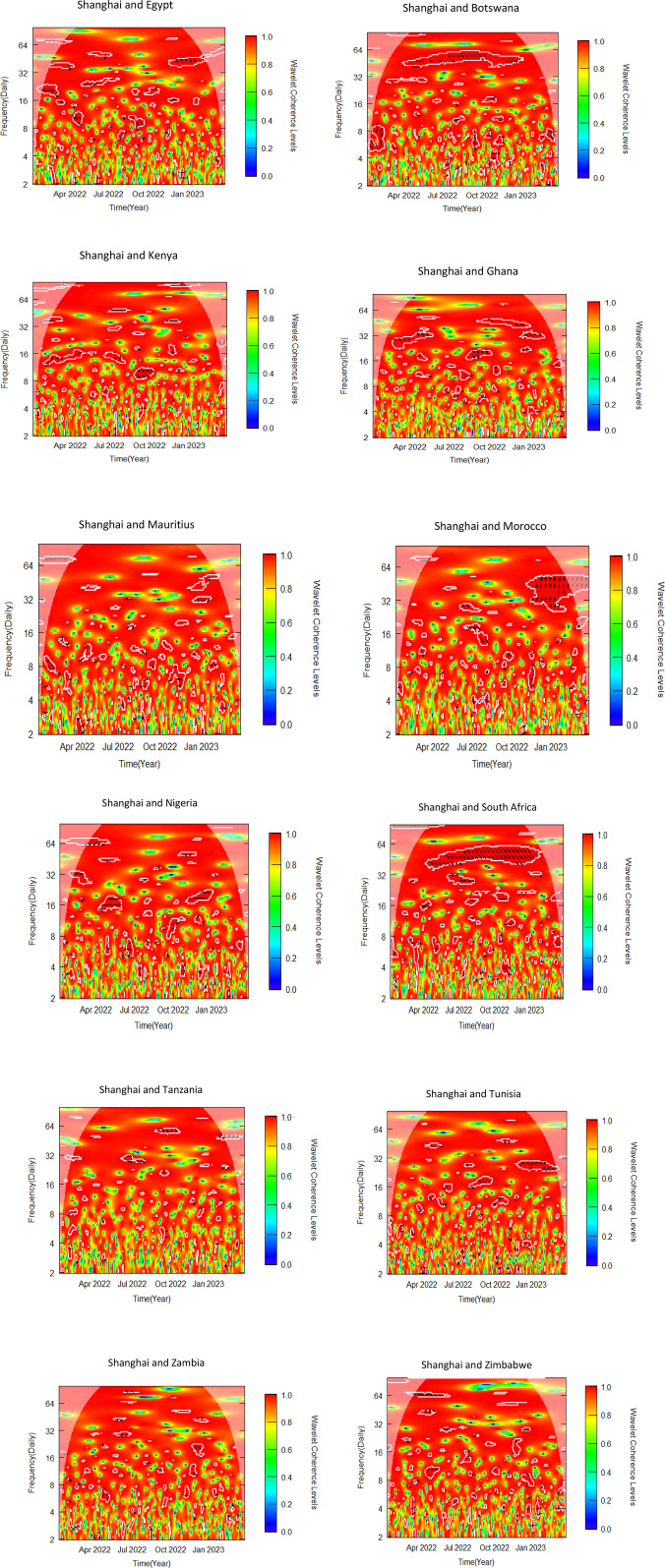
Bivariate wavelet coherence for post-COVID-19 period (Shanghai & African stock markets).

This sub-section uses bivariate wavelet coherence to examine the relationships between African stock markets and two advanced markets. The pre-COVID-19, COVID-19 era and post COVID-19 periods make up the three sub-samples from which the data is separated, as was previously indicated. To determine variations in co-movements attributable to the pandemic, the bivariate wavelet coherence is measured (Figs 2-7).

Local correlation is measured by wavelet coherence (WC), and phase-difference sheds light on any correlations between the variables. In a manner comparable to the coefficient of determination, Rösch and Schmidbauer (2018) [69] claim that WC takes into account the individual power differences and reveals the joint periodic features of the several signals.

The results from the bivariate analysis are presented in the heatmaps of Figs 2-7. Significant coherences indicate one big cone of influence (COI) and several white contours inside the COI. A strong co-movement between the two signals, either at the beginning or end of the periods, is indicated by a red colour inside the white contours [70]. Comparably, a strong co-movement at high (low) frequencies is indicated by a red colour at the top (bottom) of the heatmap.

### 4.2 Co-movement between NYSE and African stock markets in pre-COVID-19 period

Fig 2 indicates that NYSE leads Zimbabwe in-phase between 64–128 frequency bands in 2018. In the late 2018 to early 2019 NYSE leads again but out-of-phase in the same frequency band. For the NYSE-Ghana pair, NYSE leads in phase in 64−128 frequency band in 2018. NYSE-Egypt pair did not produce clear correlation except in mid-2017 where NYSE leads in phase in 64−128 frequency band. In 64 frequency band, NYSE leads Botswana in phase in early 2017. For NYSE -Tanzania pair, the arrows in the scattered contours in the COI indicate the pair were generally in phase in 2017. NYSE and South Africa are in phase in the second half of 2018. South Africa however leads NYSE out-of-phase in frequency band 64 from late 2018 to early 2019. For the NYSE-Mauritius pair, NYSE leads in phase in 64−128 frequency band in 2017. The pair are in phase in the first half of 2018. The pre-COVID-19 period shows a general influence of NYSE on stock markets in Africa. The co movement results between NYSE and most African stock markets buttress the findings of Bekaert et al. (2014) [60] which suggest that the spillover from the global financial crisis triggered co-movement of markets and therefore weakened diversification.

### 4.3 Co-movement between Shanghai and African stock markets in pre-COVID-19 period

Interestingly, Tunisia leads the Shanghai-Tunisia pair in phase from latter part of 2017 to early 2018 in frequency band 32 and 128 (Fig 3). However, in frequency 256 Shanghai leads the pair from mid-2018 to mid-2019. The Shanghai-Tanzania pair exhibited two different correlations within the period in frequency band 64–128 and with WC between 0.6 and 0.8. Shanghai leads in phase in the second half of 2017, and leads again out of phase in 2019. The WC of the Shanghai- South Africa pair seem to give a clear picture of the co-movement between the two markets. The variables are in phase over the period above 64 frequency band with South Africa leading in the latter part of 2018, and Shanghai leading in the latter part of 2019. Again, a trade off was identified between the Shanghai-Nigeria nexus. Shanghai seems to lead Nigeria out of phase briefly in 2017 in 32 frequency band, and in phase in some scattered periods between 2018 and 2019 in frequency bands 32–256. Again, Shanghai leads Kenya in phase in 32–64 frequency band with 0.6 WC in 2018. The pair were predominantly in phase in 128–256 frequency band. In 2019 the variables were in phase in frequency band 128–256. For the Shanghai-Ghana pair, the variables are in phase in frequency band 32–128. However, Shanghai leads Egypt in phase in the latter part of 2018 and 2019 in 256 frequency band. Shanghai leads Egypt in 64–128 frequency band between 2017 and early part of 2019. These co-movements buttress the interconnectedness between China and African markets. Over the past three decades, China has been building strong partnerships with African economies which have resulted in increase in trade and investment in the region [71].

### 4.4 Co-movement between NYSE and African stock markets in COVID-19 period

The co-movement between NYSE and African markets was virtually non-existent during the COVID-19-period apart from a few instances (Fig 4). In the NYSE-Tanzania pair, Tanzania leads NYSE out of phase in 8–19 frequency band with 0.4 WC. In the first part of 2021, the NYSE-Morocco pair was in phase in 32 frequency band. The situation for NYSE-Ghana pair was not significantly different. In mid-2020 and early 2021, Kenya leads out of phase in 128–32 frequencies, while NYSE leads in phase in the latter part of 2021 in 16–32 frequency band. No discernible nexus was identified between NYSE and the remaining African Markets. The findings of moderate co-movement between New York and African markets reiterate the findings of Li (2025) [72] who found that the influence of U.S. market to the global markets was greater before the COVID-19 era than during the pandemic period.

### 4.5 Co-movement between Shanghai and African stock markets in COVID-19 period

The co-movement of the Shanghai-Zimbabwe pair took different dimensions over the period. In the mid to late-2020 Zimbabwe leads in phase at 128 frequency band (Fig 5). In early to mid-2021, Shanghai leads in phase between 32 and 128 frequency band. Tunisia leads out of phase in the latter part of 2020 in the Shanghai-Tunisia pair. Tunisia leads again in phase in 64–128 frequency band just before the mid-2021. Shanghai and Tanzania trade off lead/lag in phase across board. Tunisia leads Shanghai out of phase in 16–64 frequency band from mid-2020 to early 2021. In 64–128 frequency band Shanghai leads in phase from the latter part of 2020 to early 2021. In 8–16 frequency band Tanzania leads Shanghai in phase briefly in the latter part of 2020. Shanghai-South Africa pair also trade off lead and lag nexus both in-phase and out-of-phase. Shanghai leads South Africa in phase in frequency band 64 from the latter part of 2020 to the early part of 2021. Surprisingly, in the latter part of 2021, South Africa leads in phase in 16–32 frequency band. In the Shanghai-Nigeria pair, Nigeria leads in phase in 128 frequency band from the latter part of 2020 to early 2021. In frequency band 64–128, Morocco leads in phase for the first part of 2021. Mauritius leads in phase in 128 frequency band from mid-2020 to early 2021. No discernible nexus is found Shanghai and the remaining African markets. These results support the findings of Fu et al. (2021) [73] who suggested contagion effects across global equity markets in their study on the effect of the COVID-19 epidemic on the stock markets of fifteen nations globally. The rapid spread of the pandemic and the announcement of government policies to reduce the spread of the pandemic in Africa resulted in reduction in African stock market return [74].

### 4.6 Co-movement between NYSE and African stock markets in post-COVID-19 period

Zimbabwe leads NYSE out of phase for the first part of 2022 in 32–64 frequency band (Fig 6). However, in the latter part of 2022 and early 2023 in 64 frequency band, NYSE leads the pair out of phase. In the NYSE-Tunisia pair, NYSE leads in phase for the second and third quarter of 2022 in 32–64 frequency band. However, in frequency band 8–32. Tunisia leads in phase from mid-2022 to early 2023. South Africa and NYSE are in phase in post part of the period in 32–64 frequency band. However, between 8 and 32 frequency band with 0.4–0.8 WC South Africa leads mostly in phase in the mid-2022. In mid-2022, the NYSE led Nigeria out of phase in the 32–64 frequency band, and from late 2022 to early 2023, NYSE again leads in phase in the 64 frequency band. The nexus between NYSE-Morocco pair is also varied. In the mid-2022 to the third quarter of 2022 NYSE leads in phase in 32–64 frequency band. However, Morocco leads the pair out-of-phase in late 2022 in 16 frequency band. NYSE predominantly leads Mauritius in phase throughout 2022 in 32–64 frequency band. The NYSE-Botswana pair are in phase from the beginning to mid-2022. From the mid-2022 to the end of 2022 Botswana seems to lead in phase in 8–32 frequency band as shown by two isolated contours in the middle of the COI (Fig 6). No discernible co-movement was identified between NYSE and the remaining African markets. The findings suggest that the reduction of the COVID-19 spread resulted in increased correlation between NYSE (US) and African markets [75].

### 4.7 Co-movement between Shanghai & African stock markets in post-COVID-19 period

At a lower frequency band of 4–8, Botswana leads Shanghai in-phase. Botswana leads again in phase in 32–64 frequency band from mid-2022 to early 2023. Shanghai leads Kenya in-phase in 16 frequency band from the beginning of the post-COVID-19 period to the third quarter of 2022. In October 2022, Kenya leads Shanghai in phase in 8–16 frequency band. Both Shanghai and Ghana led intermittently in phase and out of phase over the period from 16 to 64 frequency band. In the Shanghai-Morocco pair, Shanghai leads in phase from the ending of 2022 to early part of 2023. The Shanghai-Nigeria pair was in phase over the period with no market leading as indicated by the arrows in the contours in the COI (Fig 7), except in the early part of 2022 where Shanghai leads out of phase briefly. South Africa predominantly leads the Shanghai market in phase in 32–64 frequency band from mid-2022 to early 2023. In 16–32 frequency band, Tunisia leads Shanghai in phase briefly in the latter part of 2022 whilst Shanghai leads in phase in the early part of 2023. Egypt leads Shanghai in phase in 16–32 frequency band with 0.6 WC in the early part of the post-COVID period, and out of phase in the early part of 2023. No discernible nexus was identified between Shanghai and the rest of African Markets. The short to medium term co-movements between Shanghai and African markets support the findings Gu (2022) [76]. The recovery of markets after the reduction of the effect of COVID-19 resulted in gradual restoration of diversification potential between China and Africa.

In conclusion, this study shows that, over a range of time periods, advanced stock markets such as Shanghai and New York have influence on some African markets. The findings show significant co-movement between some of the twelve African markets and the two advanced markets which is in line with research findings from previous studies. The findings of this study are consistent with those of Loh (2013) [77] who used wavelet analysis to identify different co-movements across the US, European, and Asia-Pacific stock markets. The findings further support previous studies that found that the strength of co-movement differs by country and index [78]. In addition, the study reiterates the findings reported by Anyikwa and Le Roux (2020) [48] and Omane-Adjepong et al. (2020) [19] that COVID-19 had little effect on the co-movement between advanced and African markets.

The summary of significant findings of co-movement between NYSE, SSE and African stock markets indicating time horizon, direction and magnitude of co-movement as well as implications for investors and policy makers are shown in Table 6. The findings suggest significant co-movements between some African stock markets and the NYSE and SSE, suggesting that international investors seeking to construct portfolios combining African and these global markets may face limited or no opportunities for diversification. Diversification opportunities, however, exist for international investors constructing portfolios that combine African stock markets that did not report significant results with NYSE and SSE, as shown in Figs 2–7.

**Table 6:** Summary of co-movements between NYSE, SSE, and African markets and practical implications.

Time	Pairs	Leader	Phase	Direction/Magnitude	Scale	Implications (Diversification/Hedging)
Pre-COVID-19 Era						
	NYSE-Zimbabwe	NYSE	In phase	Positive/ Moderate		Limited Diversification, Hedging may be needed
2018	NYSE-Ghana	NYSE	In phase	Positive/Strong	32-128	No Diversification, Hedging needed
Early 2017	NYSE-Botswana	NYSE	In phase	Positive/ Strong	32-64	No Diversification, Hedging needed
2018–2019	NYSE- S. Africa	S. Africa	Out of Phase	Negative/Moderate	64-128	Limited Diversification, Hedging may be needed
2017 & 2019	NYSE-Morocco	Morocco	Out of phase	Negative/Moderate	32-128	Limited Diversification, Hedging may be needed
2019	SSE – S. Africa	SSE	In phase	Positive/ Moderate	32-64	Limited Diversification, Hedging may be needed
2019	SSE – Kenya	SSE	In phase	Positive/ Moderate	32-256	Limited Diversification, Hedging may be needed
2018	SSE – Ghana	SSE	In phase	Positive/Strong	64-256	No Diversification, Hedging needed
COVID-19 Era						
2020	NYSE – Kenya	Kenya	In phase	Positive/ Strong	16-128	No Diversification, Hedging needed
2021	SSE- Zimbabwe	SSE	In phase	Positive/ Strong	32-128	No Diversification, Hedging needed
2021	SSE – Tunisia	Tunisia	In phase	Positive/Strong	32-128	No Diversification, Hedging needed
2020-2021	SSE – Tanzania	SSE	In phase	Negative/Strong	8-128	No Diversification, Hedging needed
2021	SSE – S. Africa	SSE	In phase	Positive/ Strong	8-128	No Diversification, Hedging needed
2021	SSE – S. Africa	S. Africa	In phase	Positive/ Moderate	8-128	Limited Diversification, Hedging may be needed
2020	SSE – Ghana	SSE	In phase	Positive/ Strong	32-128	No Diversification, Hedging needed
2021	SSE – Nigeria	Nigeria	In phase	Positive/ Strong	32-128	No Diversification, Hedging needed
Early 2021	SSE – Morocco	Morocco	In phase	Positive/ Strong	64-128	No Diversification, Hedging needed
Post-COVID-19 Era						
2022	NYSE – Tunisia	Tunisia	In phase/	Positive/ Moderate	8-64	Limited Diversification, Hedging may be needed
2022–2023	NYSE – Tanzania	Tanzania	In phase	Positive/ Moderate	16-32	Limited Diversification, Hedging may be heeded
2022-2023	NYSE– S. Africa	S. Africa	In phase	Positive/ Moderate	8-64	Limited Diversification, Hedging may be needed
2022	NYSE – Nigeria	NYSE	In phase	Positive/ Strong	32-64	No Diversification, Hedging needed
2022	NYSE– Morocco	NYSE	In phase	Positive/ Strong	64	No Diversification, Hedging needed
2022	NYSE – Mauritius	NYSE	In phase	Positive/ Strong	32-64	No Diversification, Hedging needed
2022	NYSE– Botswana	Botswana	In phase	Positive/ Moderate	8-64	Limited Diversification, Hedging may be needed
2022–2023	SSE – Botswana	Botswana	In phase	Positive/Strong	32-64	No Diversification, Hedging needed
2022	SSE – Kenya	Kenya	In phase	Positive/ Moderate	8-16	Limited Diversification, Hedging may be needed
2022- 2023	SSE – Morocco	SSE	In phase	Positive/Strong	16-64	No Diversification, Hedging needed
2022–2023	SSE – S. Africa	S. Africa	In phase	Positive/ Strong	34-64	No Diversification, Hedging needed

### 4.8 Robustness check

The Granger Causality Wald Test results (Table 7 reveal the presence or absence of causal relationships between variables in the VAR model. Statistically significant results (p-values < 0.05) suggest that one variable Granger-causes another. Markets such as Zimbabwe (p = 0.029), South Africa (p = 0.019), Nigeria (p = 0.012), Mauritius (p = 0.000), Kenya (p = 0.000), NYSE (p = 0.000), and Shanghai (p = 0.000) demonstrate significant causality, indicating predictive relationships in these markets. In contrast, other markets, including Zambia, Tunisia, Tanzania, Morocco, Egypt, and Botswana, exhibit non-significant p-values (> 0.05), suggesting no strong causal relationships in their data. Ghana is on the cusp of significance (p = 0.058), which might warrant further investigation. These results highlight varying levels of interdependence and dynamics across markets, with global indices and certain African markets displaying stronger Granger-causal linkages.

**Table 7:** Granger Causality Wald Tests.

	chi^2^	Prob > chi^2^
Zimbabwe	41.257	0.029
Zambia	30.416	0.251
Tunisia	21.040	0.740
Tanzania	10.930	0.996
South Africa	42.994	0.019
Nigeria	44.849	0.012
Morocco	31.679	0.204
Mauritius	86.411	0.000
Kenya	62.321	0.000
Ghana	38.200	0.058
Egypt	21.579	0.711
Botswana	28.895	0.316
NYSE	83.648	0.000
Shanghai	83.952	0.000

### 4.9 Interdependence or contagion

Our findings from Fig 2-7 and captured in S1 Table, S2 Table and S3 Table, reveal that, albeit not consistently over time, interdependencies between the African stock markets and the two advanced markets were somewhat more prominent in all the three periods studied, namely pre-COVID, COVID and post-COVID-19 periods based on the description put forward by Yang et al. (2016) [43] and Forbes and Rigobon (2002) [40].

This result reiterates the findings of Beirne and Gieck’s (2012) [62] that interdependence in stock markets is most prominent between developed and emerging markets. The pairs of SSE with African markets however recorded more contagion relationship in the COVID period compared to the pre and post periods.

## 5. Conclusion, implications and recommendations

In contrast to the traditional econometric approaches used by previous scholars, this study employed the bivariate wavelet method which is particularly effective at capturing co-movements by localizing within time-frequency domains. This study deviates from most previous studies as a result of the methodology used, the markets studied and the nature of crisis analyzed - COVID-19. In addition, while previous research generally indicates that African markets have little to do with global equity markets, this research discovered co-movement between some African and advanced markets.

The findings suggest a generally consistent level of co-movement between the NYSE and SSE, and African markets across different frequencies, with notable co-movement concentrated at medium to high frequencies. The results further discover significant co-movements in the form of interdependence between the two global markets, and several African markets, underscoring that African markets are not entirely insulated from volatilities in advanced economies. Nonetheless, a substantial number of African markets exhibited insignificant co-movement with the NYSE and SSE. This weak linkage provides international investors with opportunities to diversify their portfolios by combining equities from these African markets with those from the two global markets. Such markets may also serve as potential safe havens during periods of uncertainties in advanced markets. In particular, short-term investors could exploit diversification opportunities by focusing on market pairs characterized by weak co-movement. Although some African markets displayed moderate levels of co-movement, the overall scope for international diversification remains somewhat limited.

We recommend policymakers to take into consideration the time and frequency characteristics of these markets in their decision making. Institutions and strategic investors should also take the time and frequency factors into consideration to attain maximization of returns and reduction in risk during periods of uncertainty.

Although wavelet analysis provide insight into lead/lag relationships and possible causality, more research utilizing parametric non-linear causality tests, such as those introduced by Diks and Panchenko (2006) [79], may yield more statistically significant findings over time and at different frequencies.

Future studies could build on these findings by employing more complex causality tests and using other financial assets, such as derivatives or cryptocurrencies, to determine their co-movement with assets traded on African markets during pandemics or global crises. In addition, the influence of geopolitical events and unique entropy could offer further dimensions to assimilation of stock market interdependence.

In conclusion, this research shows significance co-movements between some African markets and advanced stock markets. The assertion that African markets are insulated from global shocks is gradually fading away.

## Supporting information

S1 Table Supporting Information Table 1. (DOCX)

S2 Table Supporting Information Table 2. (DOCX)

S3 Table Supporting Information Table 3. (DOCX)

S1 File Supporting Information – File 1. (DOCX)
