# Supplementary material for: Co-movement between stock markets in advanced economies and Africa in times of uncertainty: A time-frequency domain approach
Source: PLoS One. 2025 Nov 6;20(11):e0334325. doi: 10.1371/journal.pone.0334325 (PMC12591454; doi:10.1371/journal.pone.0334325)
Supplement: S3 Table — (DOCX) [file pone.0334325.s003.docx]

| **S3 Table:**  Contagion vs Interdependence (Post-Covid)  NYSE and African Stock Markets – Post-Covid-19 Period | | | | | SSE and African Stock Markets – Post-Covid-19 Period | | | | |
| --- | --- | --- | --- | --- | --- | --- | --- | --- | --- |
| Pair | Time – Horizon | Phase (In/Out) | Lead/Cause | Relationship  (Interdependence/  Contagion) | Pair | Time - Horizon | Phase (In/Out) | Lead/Cause | Relationship  (Interdependence/  Contagion) |
| NYSE and Zimbabwe | Short term | Out of | NYSE | Interdependence | SSE and Zimbabwe | Short term | Indistinct | Indistinct | Interdependence |
|  | Med. term | Out of | Zimbabwe | Interdependence |  | Med. term | Indistinct | Indistinct | Interdependence |
|  | Long term | In | NYSE | Contagion |  | Long term | Indistinct | Indistinct | Interdependence |
| NYSE and Zambia | Short term | In | NYSE | Interdependence | SSE and Zambia | Short term | Indistinct | Indistinct | Interdependence |
|  | Med. term | Indistinct | Indistinct | Interdependence |  | Med. term | Indistinct | Indistinct | Interdependence |
|  | Long term | Indistinct | Indistinct | Interdependence |  | Indistinct | Indistinct | Indistinct | Interdependence |
| NYSE and Tunisia | Short term | Indistinct | Indistinct | Interdependence | SSE and Tunisia | Short term | Indistinct | Indistinct | Interdependence |
|  | Med. term | In | Tunisia | Interdependence |  | Med. term | In | SSE | Interdependence |
|  | Long term | In | NYSE | Interdependence |  | Long term | Indistinct | Indistinct | Interdependence |
| NYSE and Tanzania | Short term | Indistinct | Indistinct | Interdependence | SSE and Tanzania | Short term | Indistinct | Indistinct | Interdependence |
|  | Med. term | In | Tanzania | Interdependence |  | Med. term | Indistinct | Indistinct | Interdependence |
|  | Long term | Indistinct | Indistinct | Interdependence |  | Long term | Out of | Tanzania | Interdependence |
| NYSE and S. Africa | Short term | In | S. Africa | Interdependence | SSE and S. Africa | Short term | Indistinct | Indistinct | Interdependence |
|  | Med. term | In | S, Africa | Interdependence |  | Med. term | Indistinct | Indistinct | Interdependence |
|  | Long term | In | NYSE | Interdependence |  | Long term | In | S. Africa | Contagion |
| NYSE and Nigeria | Short term | Indistinct | Indistinct | Interdependence | SSE and Nigeria | Short term | Indistinct | Indistinct | Interdependence |
|  | Med. term | Indistinct | Indistinct | Interdependence |  | Med. term | Indistinct | Indistinct | Interdependence |
|  | Long term | In | NYSE | Contagion |  | Long term | Out of | SSE | Interdependence |
| NYSE and Morocco | Short term | In | NYSE | Interdependence | SSE and Morocco | Short term | Indistinct | Indistinct | Interdependence |
|  | Med. term | Out of | Morocco | Interdependence |  | Med. term | In | SSE | Interdependence |
|  | Long term | In | NYSE | Interdependence |  | Long term | Out of | SSE | Contagion |
| NYSE and Mauritius | Short term | In | Mauritius | Interdependence | SSE and Mauritius | Short term | Indistinct | Indistinct | Interdependence |
|  | Med. term | In | NYSE | Interdependence |  | Med. term | Indistinct | Indistinct | Interdependence |
|  | Long term | In | NYSE | Contagion |  | Long term | Indistinct | Indistinct | Interdependence |
| NYSE and Kenya | Short term | Indistinct | Indistinct | Interdependence | SSE and Kenya | Short term | Indistinct | Indistinct | Interdependence |
|  | Med. term | In | NYSE | Interdependence |  | Med. term | In | Kenya | Interdependence |
|  | Long term | Indistinct | Indistinct | Interdependence |  | Long term | Indistinct | Indistinct | Interdependence |
| NYSE and Ghana | Short term | Indistinct | Indistinct | Interdependence | SSE and Ghana | Short term | Indistinct | Indistinct | Interdependence |
|  | Med. term | In | NYSE | Interdependence |  | Med. term | In | Ghana | Interdependence |
|  | Long term | Indistinct | Indistinct | Interdependence |  | Long term | Out of | SSE | Interdependence |
| NYSE and Egypt | Short term | Indistinct | Indistinct | Interdependence | SSE and Egypt | Short term | In | Egypt | Interdependence |
|  | Med. term | Indistinct | Indistinct | Interdependence |  | Med. term | In | SSE | Interdependence |
|  | Long term | Indistinct | Indistinct | Interdependence |  | Long term | Out of | Egypt | Interdependence |
| NYSE and Botswana | Short term | Indistinct | Indistinct | Interdependence | SSE and Botswana | Short term | Indistinct | Indistinct | Interdependence |
|  | Med. term | In | Botswana | Interdependence |  | Med. term | Indistinct | Indistinct | Interdependence |
|  | Long term | Indistinct | Indistinct | Interdependence |  | Long term | In | Botswana | Interdependence |
