# Supplementary material for: Co-movement between stock markets in advanced economies and Africa in times of uncertainty: A time-frequency domain approach
Source: PLoS One. 2025 Nov 6;20(11):e0334325. doi: 10.1371/journal.pone.0334325 (PMC12591454; doi:10.1371/journal.pone.0334325)
Supplement: S1 File — (DOCX) [file pone.0334325.s004.docx]

**S1 File**

Data on composite stock market indices (EquityRT (https://equityrt.com/),

Data on COVID-19 (Our World in Data (https://ourworldindata.org/).
